# Phytochemical characterization of forest leaves extracts and application to control apple postharvest diseases

**DOI:** 10.1038/s41598-024-52474-w

**Published:** 2024-01-23

**Authors:** Lobna Hajji-Hedfi, Abdelhak Rhouma, Wassila Hlaoua, Kucher E. Dmitry, Ryma Jaouadi, Yosr Zaouali, Nazih Y. Rebouh

**Affiliations:** 1 Regional Centre of Agricultural Research of Sidi Bouzid, CRRA, Gafsa Road Km 6, B.P. 357, 9100 Sidi Bouzid, Tunisia; 2 Higher Agronomic Institute of Chott-Meriem, Sousse University, Sousse, Tunisia; 3 Department of Environmental Management, RUDN University, 6 Miklukho-Maklaya St., 117198 Moscow, Russian Federation; 4 Laboratory of Agricultural Production, Higher School of Agriculture of Mograne (ESAM), University of Carthage, Mograne, 1121 Zaghouane, Tunisia; 5 Laboratory of Plant Biotechnology, Department of Biology, National Institute of Applied Science and Technology, B.P. 676, 1080 Tunis Cedex, Tunisia

**Keywords:** Biological techniques, Biotechnology, Plant sciences, Environmental sciences

## Abstract

The study investigated the antifungal and phytochemical properties of three forest plants (*Eucalyptus globulus, Pistacia lentiscus,* and *Juniperus phoenicea*) against apple diseases caused by *Colletotrichum gloeosporioides* and *Alternaria alternata*. The determination of the total polyphenol and flavonoid contents in the three aqueous extracts of studied plants showed that *E. globulus* exhibited the highest contents than those of *P. lentiscus* and *J. phoenicea*. Furthermore, the three studied extracts showed very appreciable antioxidant activity with decreasing order: *E. globulus*, *P. lentiscus*, and *J. phoenicea*. The phytochemical analysis showed different common phenolic acids in the three studied plants namely: quinic acid, gallic acid, chlorogenic acid, and caffeoylquinic acid as well as other flavonoids mainly quercetin and catechin. The results of the current study demonstrated that the fungistatic activity of *E. globulus* EO (4 and 2 µl/ml) seemed to be the most effective under laboratory conditions with an inhibition zone diameter above 16 mm. However, the poisoned food technique indicated that the aqueous extract (80%) and the essential oil (4 µl/ml) of *E. globulus* exhibited the highest mycelial growth (> 67%) and spore germination (> 99%) inhibition. Preventive treatments with essential oils (4 µl/ml) and aqueous extracts (80%) applied to apple fruits inoculated with *A. alternata* and *C. gloeosporioides* resulted in the lowest lesion diameter (< 6.80 mm) and disease severity index (< 15%) and the most favorable inhibitory growth (> 85.45%) and protective potentials (> 84.92%). The results suggest that *E. globulus* has a brilliant future in the management of anthracnose and Alternaria rot of apple and provide a basis for further studies on its effects under field conditions.

## Introduction

Fungal infections are regarded as the most damaging postharvest diseases for fresh fruits, producing significant economic losses due to their negative impact on market value and fruit shelf life^1^. However, the establishment of fungal infections in fruit has therefore several consequences, ranging from yield lowering and quality depressing in the fields to retarding their nutritive value and rapid perishing after harvesting^2,3^. Furthermore, infected fruits with fungal pathogens pose an impending health risk through mycotoxins production, such as aflatoxins, ochratoxins, and fumonisin are produced by *Aspergillus* spp., *Alternaria* spp., and *Fusarium* spp. in contaminated fruits and vegetables^4^. *Alternaria* spp. produce numerous toxic metabolites such as tenuazonic acid, alternariol, alternariol methyl ether, and altenuene, which have been detected in a wide range of foods and feedstuffs infected by the pathogen. These secondary metabolites are toxic to animals, plants, and human cell cultures^5^. Tenuazonic acid is thought to be a contributing factor to onyalai, a hematological disorder in humans, and it inhibits protein synthesis^6^.

To date, fungicides are the principal methods of controlling fungal diseases^7,8^. Nevertheless, during the last few decades, their use was hampered by stricter regulatory policies limiting their doses, and their action spectrum and even restricting their use. This is due to the public rising concern about possible human health risks, undesirable environmental effects, and the development of pathogens’ resistance^9,10^.

In the absence of effective plant protection management, *Colletotrichum gloeosporioides* and *Alternaria alternata* can result in significant yield losses of up to 100% of total apple production^2,7,11^. Therefore, the development of alternative safe and natural methods for controlling fungal diseases has become an urgent need^11,12^. Substantial research, using microbial antagonists and extracts have been conducted to prove their efficacy as fungal diseases’ biological control agents and to reduce synthetic fungicides’ usage^13^. Plant extracts have also been actively explored in recent years as a potential biological agent against many postharvest diseases^9^. In this context, forest plants are also considered potent sources of phytochemicals such as phenols, flavonoids, and essential oils that could be exploited as a biological control against fungal diseases^14^.

Recently, Hajji-Hedfi et al.^15^ reported the chemical composition of aqueous extracts of *Pistacia lentiscus* and their efficacy in controlling root-knot nematodes *Meloidogyne javanica* and *Fusarium oxysporum* sp. *lycopersici*. Nevertheless, *P. lentiscus, J. phoenicea*, and *E. globulus* are considered important medicinal plants largely used in traditional medicine^16^. Indeed, numerous biological activities have been associated with *Eucalyptus* extracts, such as antibacterial, antifungal, antioxidant, herbicidal, and acaricidal activity^17^. Furthermore, the use of natural botanical ingredients is thought to be a good viable alternative to control postharvest diseases. However, few researchers have looked into the ability of plant extracts to suppress the growth of many phytopathogens^18^.

Examining numerous physicochemical properties of plant extracts reveals the usefulness of these products in daily life. Organoleptic characteristics, color, odor, density, refractive index, acid value, pH, and yield percentage are characteristics that indirectly affect the quality of essential oils^19^. These physicochemical characteristics as well as the composition, which give benchmark information to gauge an oil's appropriateness for consumption or other use, largely define the commercial importance of these products.

However, limited data are available on the biocontrol efficacy of selected plant extracts (*Eucalyptus globulus, Pistacia lentiscus,* and *Juniperus phoenicea*) against the apple rot postharvest fungal diseases and their organoleptic and phytochemical characterization.

The originality of natural plant extracts lies in their fungicidal properties, multifaceted approach, eco-friendly nature, and potential for innovative applications. By addressing existing challenges and continuing research, naturally occurring biologically active compounds from plants can pave the way for a more sustainable and effective future in plant disease management. Therefore, this study aims to determine the phytochemical composition and to evaluate the fungistatic and antioxidant activities of aqueous extracts and essential oils extracted from *E. globulus, P. lentiscus,* and *J. phoenicea*. These extracts will be tested to investigate their efficacy as new biological control sources against postharvest disease apple fruit rot caused by *C. gloeosporioides* and *A. alternata*. In addition, to the best of our knowledge, it is the first time to assess the aqueous and essential oils extracts from *E. globulus*, *P. lentiscus*, and *J. phoenicea* against *C. gloeosporioides* and *A. alternata*.

## Material and methods

### Plant material and extract preparations

Three species of forest trees, *Pistacia lentiscus* L. (Anacardiaceae), *Juniperus phoenicea* L. (Cupressaceae), and *Eucalyptus globulus* Labill. (Myrtaceae), were utilized as sources of plant material in this study. Fresh leaves were collected from the mountains of the Kairouan region in the Center East of Tunisia. Dr Aliat Taoufik from the Higher National School of Forests, Khenchela, Algeria, identified Forest plant species. No voucher specimen of these materials have been deposited in a publicly available herbarium. The plant collection complied with relevant local, institutional, national, and international guidelines, permissions, or legislation and we obtained the necessary permissions from the governorate of Kairouan. Leaves samples were carefully cleaned with tap water and then rinsed with distilled water, and shade-dried at room temperature for fifteen days. Parts of the dried leaves were ground to uniform powder using a mechanical mixer (MRC, SM 450, China). The obtained powder as well as dry leaves were stored away from moisture and light at 4 °C until further use.

#### Aqueous extracts

To prepare the aqueous extract, 10 g of the leaf powder of each plant was added to 100 ml of distilled water and vigorously mixed in an Erlenmeyer flask. The maceration process lasts 48 h at room temperature with periodic shaking every 2 h. The mixture was filtered using a Whatman N°1 filter paper to separate the aqueous extract. Different concentrations (C1: 80, C2: 60, C3: 30, C4: 20, and C5: 10% v/v) were prepared by diluting the stock aqueous solution to finalize the in vitro experiments^9^.

#### Essential oils extract

Hydro-distillation method was employed using a Clevenger-type apparatus in a 1 L flask. The extraction was performed for 2 h as follows: dry leaves of each plant (100 g) were first cut into small pieces to facilitate their placement in the round Pyrex glass flask that contains 1 L of water. Then the Pyrex glass was placed on an adjustable-temperature balloon heater. The ebullient vapor is carried via the vertical tube (rectification column) and then to the cooling column (also known as the refrigerator or serpentine) where the vapor is condensed. At the end of the hydrodistillation process, a collecting tube (settling column) collects the two liquids (essential oil and distillate). The volatile oil separates as an upper layer because it is immiscible and lighter than water. Following the separation of the oil from the water, it was gathered in small bottles, dried with anhydrous sodium sulfate, sealed, and kept in light-resistant vials at 4–6 °C for later use^1,17,20^.

#### Total polyphenols contents of aqueous extracts

The total polyphenols for the different samples were quantified using the folin-ciocalteu reagent according to the method described by Apolonio-Rodríguez et al.^20^. Briefly, 100 μl of each aqueous extract was added to 100 μl of folin-ciocalteu (1:1) and mixed. After 5 mn, 2 mL of sodium carbonate Na_2_CO_3_ (2%) was added. The mixture is vortexed and incubated for 30 min at room temperature in the dark. The volume was then adjusted to 10 ml with distilled water. Absorbance was determined, after one hour at one wavelength (750 nm) using a spectrophotometer (SP-UV5000, China). The measurement is compared to a blank and a calibration curve established by 5 increasing concentrations of gallic acid (0–1000 ppm) under the same conditions. The results were expressed as equivalent to gallic acid in milligram (EAG) mg/g of dry matter^20^.

#### Total flavonoid contents of aqueous extracts

A standard range was created based on the varying concentrations of catechin at 40, 80, 120, 160, and 200 μg/ml. For the control, 400 μL of distilled water is used. In test tubes, 400 μL of each concentration is inserted. After 5 min, 120 μL of NaNO_2_ and 120 μL of AlCl3 were added to each sample of aqueous extract as well as to the control. The mixture was vigorously stirred. 5 min after, a volume of 800 μL of NaOH was added. A spectrophotometer (SP-UV5000, China) was then used for an absorbance reading at 510 nm wavelength. The result was expressed in mg of catechin equivalents (mg CAT/g DW), through the calibration curve of the catechin (range 0–200 μg/mL)^21^.

#### Antioxidant activity of aqueous extracts

Antioxidant activity is based on the reduction of DPPH (1,1-diphény l-2-picryl-hydrazyl), a stable free radical violet in solution with a distinctive absorbance between 512 and 517 nm. DPPH was reduced to diphenylpicryl-hydrazine by a compound with anti-free radical properties. DPPH (0.0078 g) was dissolved in methanol to have a volume of 100 ml of DDPH solution. The tests were performed three times with 1 ml of DPPH solution added to 1 ml of extracts. The absorbance was measured against a blank at 517 nm (SP-UV5000 spectrophotometer, China). The inhibition activity of DPPH radical of the studied extracts is then expressed in % and calculated according to the following formula (1) ^20^:

1 $${\text{Inhibition}}\;{\text{activity}}\left( \% \right) = \left[ {\left( {{\text{Ac}} - {\text{Ae}}} \right)/{\text{Ac}}} \right]*{1}00$$

where Ac is the absorbance of the control of DPPH, and Ae is the absorbance of the sample in the presence of DPPH.

#### Phenolic profile of aqueous extracts by HPLC–DAD

The phenolic compounds were quantified using a Shimadzu UFLC XR system (Kyoto, Japan), equipped with a SIL-20 AXR auto-sampler, a CTO-20 AC column oven, an LC binary pump-20ADXR and a 2020 quadruple detector system.

In this part of the study, we used the aqueous extracts (previously prepared), and separation was carried out by HPLC (high-performance liquid chromatography). The combinations of these different products (H_2_O (95%), methanol (5%), acetic acid (0.15%), and (acetonitrile (50%), H_2_O (50%), folic acid (0.1%) have been used as mobile phases A and B, respectively. Phenolic compounds were identified by comparison with the retention time of phenolic standards and results were reported as mean ± standard error^22^.

#### Organoleptic and physicochemical properties of essential oils

A benchmark for the quality of oils is provided by their physicochemical properties. General aspects of essential oils’ physicochemical properties were determined. Color and odor were scored using a scale from 1 to 8; 1 = extremely weak intensity, 2 = very weak intensity, 3 = weak intensity, 4 = moderate intensity, 5 = slight intensity 6 = strong intensity, 7 = very strong intensity, 8 = extremely strong intensity^23,24^. pH using a pH meter (HI 9125-Hanna-USA), density according to AFNOR. NF ISO 279, (Standard NFT 75–111)^25^, refractive index using an Atago (Atago pal-α, Japan)^26^, acid index by referring to ISO 1242:1999/NF ISO 1242:1999 (T 75–103)^27^, and percentage of yield, which was determined about the dry mass (100 g) of the original sample.

#### Essential oils composition: gas chromatography analyses

GC–MS analyses were performed with a gas chromatograph (Agilent 7890A) equipped with an HP-5MS capillary column (30 m × 0.25 mm) and associated with a mass selective detector (Agilent 5975C inter MSD). The flow of the carrier gas (helium) was 0.8 ml/min. The oven temperature was programmed from 60 to 240 °C at 4 °C/min. The injector temperature was maintained at 250 °C. The temperatures of the quadrupole and the source were 150 and 230 °C, respectively. The mass scan ranged from 50 to 550 m/z at 70 eV. Essential oil components were identified by comparison of their retention times with those of authentic standards available in the laboratory of plant biotechnology, National Institute of Applied Science and Technology (Tunis, Tunisia), and also by comparison of their retention indices according to the literature. The retention indices were calculated according to a series of n-alkanes (C9–C24). The identification was also completed by comparison of their mass spectra with those stored in NIST08 and W8N08 libraries^28^.

### Fungal strains

*Alternaria alternata* (Fr. keissl.) (Pleosporaceae) and *Colletotrichum gloeosporioides* (Penz.) (Glomerellaceae), causal agents of apple rot, used in the present study were obtained from the laboratory of Plant Protection and Biological Sciences, Higher Agronomic Institute of Chott-Meriem (Sousse, Tunisia). These phytopathogens were isolated from infested apple fruits (cv. Zina) collected from fields cultivated with apple trees (Sbiba, Kasserine, Tunisia).

#### Fungistatic activity of essential oils

The relative efficacy of essential oils on inhibition zone diameter (IZD) of *A. alternata* and *C. gloeosporioides* was studied in vitro using the disc diffusion method, as described by Perczak et al.^29^.

Fungistatic activity is revealed by the absence or presence of mycelia proliferation. It results in a translucent halo around the disc identical to sterile agar^30,31^.

#### Effect of essential oils and aqueous extract on mycelium growth and spore germination inhibition

The effect of *E. globulus, J. phoenicea*, and *P. lentiscus* essential oils on the spore germination of the pathogenic fungi was tested in potato dextrose broth (PDB) as described by Moreira et al.^32^ and Droby et al.^33^. The percentage of inhibition of spore germination was estimated using the formula (2):

2 $$\% {\text{SIG}} = \left( {\left( {{\text{C}} - {\text{D}}} \right)/{\text{C}}} \right)*{1}00$$

where C, Number of germinated spores in the control treatment, and D, Number of germinated spores in the experimental treatment.

Different concentrations of aqueous extracts (10%, 20%, 30%, 60% and 80%) and essential oils (4, 2, 1, 0.5, 0.25 and 0.125 µl/ml PDA) containing 0.05% dimethylsulfoxide (DMSO) each were prepared to evaluate their fungistatic activities against *A. alternata* and *C. gloeosporioides* according to the method of poisoned food as described by Matrood and Rhouma^9^. Then, the results were expressed as percent inhibition of radial growth using the formula (3) Philippe et al.^34^:

3 $${\text{Pl}}\left( \% \right) = \left( {\left( {{\text{D}} - {\text{d}}} \right)/{\text{D}}} \right)*{1}00$$

where: Pl is the percentage inhibition, D is the mycelial growth in control Petri dishes, and d is the mycelial growth in the test Petri dishes.

A resumption of growth indicates a fungistatic effect and the absence of growth has a fungicidal effect.

#### Minimum inhibitory concentration

The minimum inhibitory concentration (MIC) is the lowest concentration of tested plant extract (essential oil or aqueous extract) that completely inhibits the growth of *A. alternata* and *C. gloeosporioides*^35^.

### In vivo fungistatic activity assay

The in vivo assay was conducted as previously described by Zhao et al.^36^. Healthy apple fruit cv. ‘Zina’(without physical injuries and infections) were harvested from fields cultivated with apple trees (Sbiba, Kasserine, Tunisia) based on size uniformity. Sampling fruits were soaked in 2% sodium hypochlorite for 3 min, then washed twice with distilled water, and air-dried. Apple fruits were wounded with a sterilized cork borer (6 mm diameter and 5 mm depth), for three wounds per fruit. Then, the fruits were dipped separately in prepared essential oils (4, 2, 1, 0.5, 0.25, and 0.125 µl/ml) and aqueous extracts (80, 60, 30, 20, and 10% v/v) concentrations of *E. globulus*, *P. lentiscus*, and *J. phoenicea* for 5 min. Two hours later, treated fruits were inoculated separately by spraying with 50 μL of *A. alternata* and *C. gloeosporioides* spore suspensions (10^6^ spores.mL^−1^). Two controls were performed; one by inoculating the fruits with pathogen only (positive control), and the other with distilled water (negative control). Each treatment consisted of 27 fruits per replicate (3 replicates), and the experiment was performed twice. An average of nine treated fruits were placed in plastic containers on sterile wet paper. The containers were enclosed in a plastic bag to maintain high humidity (> 90%) and subsequently incubated for 7 days in a growth chamber at 25°C^36^.

After the incubation time, the lesion diameter (LD) (mm) and disease severity index (DSI) (%) were assessed. McKinney’s equation was used to calculate the disease severity index: DSI (%) = (Σvn)/(NV) × 100, where v represents the numeric value of the disease index (DI) scale, n is the number of plants assigned to the disease index scale, N is the total number of the plants and V is the numeric value of the highest disease index scale^9^. The disease index (DI) with a scale from 0 to 5; 0 = no rotting, 1 = 1–10%of the fruit surface, 2 = 11–20%of the fruit surface, 3 = 21–30%of the fruit surface, 4 = 31–50%of the fruit surface, and 5 = more than 50%of the fruit surface^37^. The efficacy of each treatment was rated based on its DSI: EE: Extremely effective (DSI = 0%), HE: Highly effective (DSI = 0.1–5%), E: effective (DSI = 5.1–25%), I: In-effective (DSI = 25.1–50%) and HI: Highly in-effective (DSI = 50.1–100%)^38^.

To compare the treatment’s effectiveness, the percentage values of infected wounds were transformed into percentages of protective (PP) and inhibitory growth potential (IGP), as follows^39^:

4 $${\text{Protective}}\;{\text{potential}}\left( {{\text{PP}}} \right) = {1}00 \times \left[ {\left( {{\text{DSI}}_{{{\text{positive}}\;{\text{control}}}} - {\text{DSI}}_{{{\text{treated}}\;{\text{fruit}}}} } \right)/\left( {{\text{DSI}}_{{{\text{positivecontrol}}}} } \right)} \right]$$

5 $${\text{Inhibitory}}\;{\text{growth}}\;{\text{potential}}\left( {{\text{IGP}}} \right) = {1}00 \times \left[ {\left( {{\text{LD}}_{{{\text{positive}}\;{\text{control}}}} - {\text{LD}}_{{{\text{treated}}\;{\text{fruit}}}} } \right)/\left( {{\text{LD}}_{{{\text{positive}}\;{\text{control}}}} } \right)} \right]$$

### Statistical analysis

Each tested concentration of essential oils and aqueous extracts of the studied plants is tested three times. The fungistatic activity of both products was also repeated 3 times (n = 9), and the average of the tests is considered for statistical analysis. The variance analyses (ANOVA) are performed with the statistical software SPSS (version 20) to compare the effect of the different studied concentrations. For each case, the differences are considered significant at 5% (*P* ≤ 0.05) according to Tukey's multiple-range test.

### Ethics declarations

The experiments did not involve endangered or protected species. All plant materials were collected and used in accordance with national and international standards and local laws and regulations. Furthermore, the sites where the plant material was harvested are not included in national or local parks or any other natural protected areas.

## Results

### Phytochemical screening of aqueous extracts

The phytochemical and bioactive properties of the aqueous extracts of studied plant species clearly show that *E. globulus* has the highest contents of total polyphenols (TPC) (348.17 mg EAG/g DW) compared to *P. lentiscus* and *J. phoenicea* which record respectively the following amount (319.88 mg EAG/g DW), (260.69 mg EAG/g DW) (Table 1).

**Table 1:** Phytochemical screening of total polyphenol content (TPC), total flavonoid content (TFC), antioxidant capacity, and content of phenolic constituents in dried leaves of three forest trees.

Name	Forest plant species		
	*E. globulus*	*P. lentiscus*	*J. phoenicea*
TPC (mg GAE/g D.W.)	348.17 ± 1.62	319.88 ± 1.56	260.69 ± 1.01
TFC (mg Cat.E/ g D.W.)	71.56 ± 0.42	65.23 ± 1.60	72.82 ± 1.35
DPPH radical scavenging activity (%)	78.65 ± 1.83	58.78 ± 1.28	48.84 ± 0.66

Phenolic constituent	Concentration (ppm)		
Quinic acid	5464.09 ± 1.44	28,258.90 ± 1.70	6811.62 ± 1.92
Gallic acid	259.45 ± 1.18	459.55 ± 1.47	0 ± 0.00
Protocatchuic acid	0 ± 0.00	89.18 ± 1.33	12.47 ± 1.72
Chlorogenic acid	141.27 ± 1.21	0.86 ± 0.02	0.57 ± 0.07
4-O-caffeoylquinic acid	55.35 ± 1.81	0.26 ± 0.03	0.15 ± 0.03
3.4-di-O-caffeoyquinic acid	0.38 ± 0.01	0 ± 0.00	1.55 ± 0.06
1.3-di-O-caffeoyquinic acid	2.65 ± 0.01	0 ± 0.00	0 ± 0.00
Caffeic acid	4.80 ± 0.58	0 ± 0.00	0 ± 0.00
p-coumaric acid	16.34 ± 0.59	16.45 ± 0.72	0 ± 0.00
Syringic acid	106.03 ± 1.62	0 ± 0.00	0 ± 0.00
Transfrulic acid	33.17 ± 1.67	0 ± 0.00	0 ± 0.00
Hyperoside (quercetin-3-o-galactoside)	63.68 ± 0.60	1864.89 ± 1.91	38.77 ± 0.72
quercetin-3-o-rhamonosid	116.89 ± 1.17	564.05 ± 1.64	50.12 ± 0.66
quercetin	326.04 ± 1.72	90.57 ± 1.23	7.45 ± 0.45
Catechin ( +)	3.14 ± 0.43	86.06 ± 0.69	1013.98 ± 1.73
Epicatechin	0.00 ± 0.00	3.14 ± 0.57	60.71 ± 1.49
Rutin	963.74 ± 1.76	26.18 ± 0.88	0.00 ± 0.00
Naringin	0 ± 0.00	1.38 ± 0.09	1.77 ± 0.02
Naringenin	0 ± 0.00	8.62 ± 0.09	5.37 ± 0.46
Apegenin-7-o-glucoside	0 ± 0.00	162.61 ± 1.40	7.32 ± 017
Apegenin	2.83 ± 0.07	47.84 ± 0.64	6.62 ± 0.28
kampherol	0.62 ± 0.04	96.16 ± 0.66	0.07 ± 0.00
Luteolin	5.65 ± 0.33	0 ± 0.00	4.76 ± 002
Cirsiliol	22.31 ± 0.29	10.10 ± 0.14	5.58 ± 0.33
Cirsilineol	4.46 ± 0.10	0 ± 0.00	5.58 ± 0.33
Acacetin	0 ± 0.00	0 ± 0.00	8.69 ± 0.27

Means ± standard error; the data are the average of 3 samples of each essential oil per replicate (with 3 replicates); nd: not determined. mg GAE/g D.W.: mg of gallic acid equivalent per g of dry weight; mg Cat. E/ g D.W.: mg of catechin equivalents per g of dry weight.

The flavonoid contents in three aqueous plant extracts were determined to be in the range of 71.56 Ec/g DW for *E. globulus*, 65.23 mg Ec/g DW for *J. phoenicea*, and 72.82 Ec/g DW for *P. lentiscus* (Table 1).

The obtained results showed that plant extracts have high antioxidant activity. The anti-radical capacities recorded in our study are of decreasing order: 78.65% for *E. globulus*, followed by *P. lentiscus* records a value of 58.78% and 48.84% for *J. phoenicea* (Table 1).

Analysis of phenolic constituents of the leaf's aqueous content shows some variations by plant species (Table 1). According to the results, phenolic acids (quinic acid, gallic acid, chlorogenic acid, and caffeoylquinic acid) dominate the composition. The coumaric acid was only present in the extract of *E. globulus*, and *P. lentiscus*, while the synergic, and transfrulic acid were only detected in the extract of the Eucalyptus plant (Table 1).

The main detected flavonoids in our plants are quercetin, rutin, and catechin. Quercetin-3-o-galactoside is the most concentrated flavonoid in *P. lentiscus* extract (1864.89 ppm), followed by catechin (1013.98 ppm) in *J. phoenicea* and rutin (963.74 ppm) in *E. globulus* extract. It is to be noted that *E. globulus* extract included the most important number of components by its aqueous extract. The main detected flavonoids in the extract of *P. lentiscus* classified according to their concentration in the extract are as follows: quercetin, apeginin, kampherol, catechin, rutin, cirsiliol, and naringenin (Table 1).

### Phytochemical characterization of essential oils

The organoleptic and physicochemical results of essential oils are shown in Table 2. All three plants produced liquid essential oils with the yellow color varying from very light for the *J. phoenicea* extract to darker coloration for *P. lentiscus* and greenish–yellow pigmentation for *E. globulus*. The different extracts presented pleasant odors with fresh and strong fragrances for *E. globulus* and *J. phoenicea* respectively whereas smelled a strong spicy flavor (Table 2).

**Table 2:** Organoleptic and physicochemical properties of essential oils of *Pistacia lentiscus*, *Juniperis phoenicea*, and *Eucalyptus globulus* obtained by hydro-distillation extraction.

Proprieties	Plant leaves origin			*P*-value^b^
	*Eucalyptus globulus*	*Pistacia lentiscus*	*Juniperus phoenicea*	
Organoleptic				
Aspect	Liquid	Liquid and limpid	Liquid	nd
Color	Yellow, green	dark yellow	Very light yellow	nd
Smell	Fresh	strong, spicy flavor	strong	nd
Physicochemical				
pH	4.26 ± 0.021^a^	4.29 ± 0.011	4.28 ± 0.010	≥ 0.05
Density (g/cm^3^)	0.879 ± 0.005	0.877 ± 0.005	0.834 ± 0.002	≥ 0.05
Refractive index	1.468 ± 0.005	1.469 ± 0.002	1.479 ± 0.003	≥ 0.05
Acid index (mgKOH/g)	2.84 ± 0.002	1.87 ± 0.005	1.23 ± 0.010	< 0.01
Yield (%)	3.14 ± 0.04	0.1 9 ± 0.08	0.58 ± 0.17	< 0.01

Means ± standard error; ^a^Tukey’s Test; the values followed by the various superscripts differ significantly at *P* ≤ 0.05; ^b^Probabilities associated with individual F tests; nd: not determined; the data are the average of 3 samples of each essential oil per replicate (with 3 replicates).

The obtained physicochemical parameters demonstrated an acid pH of the three extracts, a close purity presented by the refraction index of about 1, 4, and a similar density value ranging from 0.87 g/cm^3^ for *E. globulus* and *P. lentiscus* to 0.83 g/cm^3^ for *J. phoenicea*. The acid index representing the stability degree of the essential oil was significantly different between the three species and the most important 2.84 mg KOH/g was that of *E. globulus* which by the way gave the best extraction yield of 3.14% by hydro-distillation (Tables 2 and 3).

**Table 3:** Effect of three essential oils at different concentrations on sensory scores (color, odor).

Forest species	Essential oil concentrations (µl/ml)	Color	Odor
*E. globulus*	4	5.33 ± 0.05a^a^	7.00 ± 0.89a
	2	4.33 ± 0.04a	5.00 ± 0.32b
	1	2.67 ± 0.07b	2.33 ± 0.08c
	0.5	2.00 ± 0.11bc	2.00 ± 0.18 cd
	0.25	1.33 ± 0.08c	1.33 ± 0.06 cd
	0.125	1.00 ± 0.00c	1.00 ± 0.00d
*P*-value^b^		< 0.01	< 0.01
*P. lentiscus*	4	6.00 ± 0.19a	4.33 ± 0.25a
	2	5.00 ± 0.13ab	2.00 ± 0.07b
	1	4.33 ± 0.09bc	1,33 ± 0.09b
	0.5	3.33 ± 0.03 cd	1.00 ± 0.00b
	0.25	2.33 ± 0.11de	1.00 ± 0.00b
	0.125	1.33 ± 0.01e	1.00 ± 0.00b
*P*-value		< 0.01	< 0.01
*J. phoenicea*	4	4.33 ± 0.12a	6.00 ± 0.71a
	2	3.33 ± 0.19b	4.67 ± 0.09b
	1	2.33 ± 0.22c	1.67 ± 0.13c
	0.5	1.33 ± 0.01d	1.00 ± 0.00c
	0.25	1.00 ± 0.00d	1.00 ± 0.00c
	0.125	1.00 ± 0.00d	1.00 ± 0.00c
*P*-value^b^		< 0.01	< 0.01

Means ± standard error; ^a^Tukey’s Test; the values followed by the various superscripts differ significantly at *P* ≤ 0.05; ^b^Probabilities associated with individual F tests; nd: not determined; The data are the average of 3 samples of each essential oil per replicate (with 3 replicates); 1 = extremely weak intensity, 2 = very weak intensity, 3 = weak intensity, 4 = moderate intensity, 5 = slight intensity 6 = strong intensity, 7 = very strong intensity, 8 = extremely strong intensity.

The chemical composition of different extracts is presented in Table 4. The most common class of chemical compounds identified were sesquiterpenes hydrocarbons and monoterpenes hydrocarbons, while the esters were the lowest (Table 4). Results show that extracts are mainly composed of terpenoids and organic compounds witch concentrations vary between the three studied plants. According to the results, each plant EO extract is characterized by some specific components. For example, *P. lentiscus* presented four specific constituents, which are Terpinen-4-ol, β-Gurjunene, Epicadinol, and Isoledene. *J. phoenicea* displayed six distinct components, while *E. globulus* had nine unique ones (Table 4).

**Table 4:** Mean percentage of the essential oil compounds at the species levels.

Chemical compounds	Class of compounds	RT	*P. lentiscus*	*J. phoenicea*	*E. globulus*
Methyl isoamyl acetate	E	4.58	2.24 ± 0.37	0 ± 0.00	10.52 ± 0.45
α-Pinene	MH	5.41	2.2 ± 0.13	48.43 ± 1.88	5.64 ± 0.16
β-Pinene	MH	6.434	0 ± 0.00	0.71 ± 0.35	0.27 ± 0.01
β-Myrcene	MH	6.72	1.55 ± 0.04	1.92 ± 0.05	0 ± 0.00
3-Carene	MH	7.275	0 ± 0.00	4.59 ± 0.21	0 ± 0.00
10-Thujene	MH	7.813	0 ± 0.00	13.07 ± 0.49	0 ± 0.00
Limonene	MH	7.87	31.60 ± 0.8	0 ± 0.00	0 ± 0.00
Eucalyptol	E	7.991	0 ± 0.00	0 ± 0.00	34.79 ± 0.46
ɣ-Terpinene	MH	8.689	0 ± 0.00	0 ± 0.00	0.44 ± 0.04
Linalool	MO	9.91	2.73 ± 0.01	0.35 ± 0.35	0 ± 0.00
Trans-pinocarveol	MH	11.2	1.58 ± 0.03	0 ± 0.00	3.24 ± 0.01
Pinocarvone	MO	11.933	0 ± 0.00	0 ± 0.00	0.49 ± 0.05
Terpinen-4-ol	MO	12.44	2.36 ± 0.09	0 ± 0.00	0 ± 0.00
Carvomenthenol	MO	12.45	0 ± 0.00	0 ± 0.00	1.39 ± 0.07
α-Terpineol	MO	12.91	2.37 ± 0.05	1.85 ± 0.33	0.79 ± 0.02
β-Citronellol	MO	14.073	0 ± 0.00	2.33 ± 0.67	0 ± 0.00
Bornyl acetate	MH	15.91	1.31 ± 0.08	0.00 ± 0.00	0 ± 0.00
4-Carene	MH	17.98	0.40 ± 0.11	7.46 ± 0.22	0 ± 0.00
α-Gurjunene	SH	19.932	0 ± 0.00	0 ± 0.00	0.67 ± 0.01
β-Caryophyllene	SH	20.26	1.60 ± 0.02	0.88 ± 0.44	0 ± 0.00
Aromadendrene	SH	20.87	3.47 ± 0.03	0 ± 0.00	7.97 ± 0.04
ɣ-Muurolene	SH	22.021	1.43 ± 0.03	0 ± 0.00	0 ± 0.00
D-Germacrene	SH	22.18	3.95 ± 0.08	0.76 ± 0.38	0 ± 0.00
β-Gurjunene	SH	22.59	1.66 ± 0.12	0 ± 0.00	0 ± 0.00
Calamenene	SH	23.417	0 ± 0.00	2.65 ± 0.05	0 ± 0.00
δ-Cadinene	SH	23.42	4.21 ± 0.06	2.58 ± 0.7	0 ± 0.00
Ledene	SH	24.229	0 ± 0.00	1.17 ± 0.7	2.02 ± 0.05
B-Germacrene	SH	24.436	0 ± 0.00	1.31 ± 0.66	0 ± 0.00
Epiglobulol	SH	24.57	2.49 ± 0.03	0 ± 0.00	3.34 ± 0.07
ɣ-Gurjunene	SH	25.34	17.60 ± 0.25	4.46 ± 1.39	14.41 ± 0.19
Viridiflorol	SH	25.54	4.07 ± 0.05	0 ± 0.00	0 ± 0.00
δ-Gurjunene	SH	25.597	0 ± 0.00	0 ± 0.00	5.04 ± 0.09
β-Eudesmol	MO	25.837	0 ± 0.00	0 ± 0.00	2.03 ± 0.05
Eremophilene	SH	26.175	0 ± 0.00	0 ± 0.00	2.01 ± 0.02
Naphthalene	SH	26.507	0 ± 0.00	3.57 ± 0.96	0 ± 0.00
Epicadinol	SH	26.93	2.51 ± 0.46	0 ± 0.00	0 ± 0.00
Isoledene	SH	27.302	3.98 ± 0.36	0 ± 0.00	0 ± 0.00
Total identified compounds	Nd	Nd	95.40 ± 0.62	98.09 ± 0.72	95.21 ± 0.49

RT, Retention time; E, Esters; MH, Monoterpenes hydrocarbons; MO, Monoterpenes oxygenated; SH, Sesquiterpenes hydrocarbons.

### Fungistatic activity of essential oil

The disc diffusion method was used to emphasize the fungistatic potency of essential oils on the development of the studied fungus species. The results indicate that the essential oil of *E. globulus* has the potential to prevent the mycelial development of (*A. alternata and C. gloeosporioides*). Both fungi were classified as sensitive toward the essential oil of *E. globulus* (Table 5). At the concentration C1 (½), all plants showed potency against both pathogenic fungi with suppressive zone diameters between 15.12 and 22.65. This inhibition was confirmed by microscopic observation of the spore suspensions with and without essential oil disk (Table 5).

**Table 5:** Inhibition Zone Diameter (IZD) of essential oils of some forest species against post-harvest apple pathogenic fungi using agar well diffusion assay.

Plant species	Essential oil concentrations	IZD Phytopathogenic fungi (mm)	
		*C. gloeosporioides*	*A. alternata*
*E. globulus*	4	16.34 ± 0.01a^a^*	22.65 ± 0.21a
	2	13.22 ± 0.23b	18.67 ± 0.09b
	1	11.15 ± 0.27c	16.12 ± 0.11c
	0.5	9.22 ± 0.33d	12.15 ± 0.14d
	0.25	6.18 ± 0.11e	7.23 ± 0.13e
	0.125	00.00 ± 0.00f	00.00 ± 0.00f
*P. lentiscus*	4	12.23 ± 0.04a	16.56 ± 0.12a
	2	9.45 ± 0.06b	14.38 ± 0.08b
	1	8.32 ± 0.05c	14.11 ± 0.10b
	0.5	8.22 ± 0.09c	10.26 ± 0.21c
	0.25	7.11 ± 0.12d	9.45 ± 0.13d
	0.125	00.00 ± 0.00	00.00 ± 0.00e
*J. phoenicea*	4	13.56 ± 0.40a	15.12 ± 0.04a
	2	9.11 ± 0.09b	9.65 ± 0.06b
	1	5.10 ± 0.08c	7.81 ± 0.02c
	0.5	3.00 ± 0.02d	5.12 ± 0.03d
	0.25	00.00 ± 0.00e	1.51 ± 0.01e
	0.125	00.00 ± 0.00e	00.00 ± 0.00e
*P*-value^b^		< 0.01	< 0.01
DMSO (negative control)		–	–

Values are mean ± standard error of the mean for bioassay conducted in triplicates. *Sensitive (IZD ≥ 11 mm: Bauer et al., 1966); ^a^ Tukey’s Test, values followed by different superscripts are significantly different at *P* ≤ 0.05; ^b^ Probabilities associated with individual F tests.

### Effect of essential oils and aqueous extract on mycelium growth and spore germination inhibition

The effects of essential oils extracts of forest plant leaves at different dilutions on the spore germination and mycelium growth of *C. gloeosporioides* and *A. alternate* are shown in Table 6. The results showed significant inhibition of fungal spore germination (*P* < 0.05) by *J. phoenicea* followed by *P. lentiscus* and *E. globulus* at different concentrations. Data recorded after 7 days revealed that *J. phoenicea* EOs was most effective in inhibiting fungal spore germination from 50 to 100% at all applied concentrations (4, 2, 1, 0.5, and 0.25 µl/ml) (Table 6).

**Table 6:** Mycelial growth Inhibition (%) and spore germination (%) rates of forest dried leaves essential oils against *A. alternata* and *C. gloeosporioides* after seven days of treatment.

Plant species	Essential oil concentrations (µl/ml)	Mycelial growth inhibition (%)		Spore germination inhibition (%)	
		*C. gloeosporioides*	*A. alternata*	*C. gloeosporioides*	*A. alternata*
*E. globulus*	4	78.37 ± 0.02a^a^	72.86 ± 0.22a	100.00 ± 0.00a	100.00 ± 0.00a
	2	78.34 ± 0.01a	72.65 ± 0.21a	100.00 ± 0.00a	100.00 ± 0.00a
	1	63.22 ± 0.23ab	58.67 ± 0.09b	98.57 ± 0.05a	81.14 ± 1.50a
	0.5	31.15 ± 0.24 c	36.12 ± 0.11c	75.14 ± 0.01b	45.71 ± 1.93b
	0.25	19.12 ± 0.23 d	12.15 ± 0.14 d	49.57 ± 0.02c	32.57 ± 1.20b
	0.125	6.16 ± 0.01 d	7.23 ± 0.13d	31.42 ± 0.02c	22.58 ± 1.02c
*P*-value^b^		< 0.01	< 0.01	< 0.01	< 0.01
*P. lentiscus*	4	71.23 ± 0.04a	62.23 ± 0.04a	98.57 ± 0.01a	95.24 ± 0.02a
	2	69.23 ± 0.04a	56.56 ± 0.12ab	95.00 ± 0.03a	94.08 ± 0.01a
	1	68.45 ± 0.06ab	49.38 ± 0.08	93.55 ± 0.09a	88.51 ± 0.08a
	0.5	28.32 ± 0.04c	24.11 ± 0.10c	76.16 ± 0.02b	65.14 ± 0.01ab
	0.25	18.2 ± 0.06c	12.26 ± 0.21c	58.75 ± 0.04b	49.27 ± 0.02b
	0.125	8.11 ± 0.12d	5.45 ± 0.13d	42.32 ± 0.03c	31.41 ± 0.02c
*P*-value		< 0.01	< 0.01	< 0.01	< 0.01
*J. phoenicea*	4	52.23 ± 0.04a	53.23 ± 0.04a	100.00 ± 0.00a	100.00 ± 0.00a
	2	43.56 ± 0.40ab	45.82 ± 0.04b	99.00 ± 0.02a	98.05 ± 0.05a
	1	39.11 ± 0.09b	39.67 ± 0.06c	98.05 ± 0.04a	88.57 ± 0.10ab
	0.5	25.10 ± 0.08c	27.83 ± 0.02c	84.19 ± 0.01b	78.14 ± 0.01b
	0.25	13.00 ± 0.02d	15.12 ± 0.03d	51.52 ± 0.02c	49.52 ± 0.02c
	0.125	00.00 ± 0.00e	1.52 ± 0.07d	38.42 ± 0.02c	33.34 ± 0.02c
*P*-value^b^		< 0.01	< 0.01	< 0.01	< 0.01

Values are mean ± standard error of the mean for bioassay conducted in triplicates; ^a^ Tukey’s Test, values followed by different superscripts are significantly different at *P* ≤ 0.05; ^b^ Probabilities associated with individual F tests.

Regarding the fungal mycelium growth, *E. globulus* was the most efficient in inhibiting *C. gloeosporioides* and *A. alternata* growth by recording inhibition rates of 78.37% and 72.86%, respectively. The last *J. phonenicea* extracts are shown to be the least effective one in reducing in vitro mycelium growth of both fungi (Table 6).

*C. gloeosporioides* and *A. alternata* were exposed to different concentrations (C1–C5) of three aqueous extracts, after 7 days the mycelia growth of both fungi was measured and the spores’ germination rates were controlled (Table 7). Obtained results show that the inhibition of the mycelial growth of the fungus gradually increases with the concentration of the extract and reaches about 67% for *C. gloeosporioides* with the most concentrated extract “C5” (80% v/v) (Table 7).

**Table 7:** Inhibition rates (%) of mycelial growth and spore germination of forest dried leaves aqueous extracts against two post-harvest fungal pathogens species after seven days of incubation.

Forest species	Aqueous extracts concentrations	Mycelial growth inhibition (%)		Spore germination inhibition (%)	
		*C. gloeosporioides*	*A. alternata*	*C. gloeosporioides*	*A. alternata*
*E. globulus*	80%	67 ± 0.04a^a^	80 ± 0.09a	100 ± 0.00a	99.33 ± 0.03a
	60%	64 ± 0.07b	59 ± 0.10b	92.67 ± 0.01b	94.67 ± 0.11a
	30%	54 ± 0.07c	49 ± 0.02c	56 ± 0.12c	44 ± 0.15b
*E. globulus*	80%	67 ± 0.04a^a^	80 ± 0.09a	100 ± 0.00a	99.33 ± 0.03a
	60%	64 ± 0.07b	59 ± 0.10b	92.67 ± 0.01b	94.67 ± 0.11a
	30%	54 ± 0.07c	49 ± 0.02c	56 ± 0.12c	44 ± 0.15b
	20%	33.38 ± 0.08d	38 ± 0.06d	45.33 ± 0.22d	36 ± 0.37c
	10%	13.68 ± 0.11e	22 ± 0.01e	26.33 ± 0.17e	17.33 ± 0.19d
*P*-value^b^		< 0.01	< 0.01	< 0.01	< 0.01
*P. lentiscus*	80%	62 ± 0.09a	52 ± 0.04a	97.67 ± 0.08a	95.67 ± 0.11a
	60%	53 ± 0.07b	52 ± 0.09a	93 ± 0.06a	92.33 ± 0.13a
	30%	51 ± 0.11b	50 ± 0.05a	55.67 ± 0.08b	53.33 ± 0.17b
	20%	22 ± 0.18c	34 ± 0.02b	41 ± 0.05c	43.67 ± 0.22c
	10%	15 ± 0.22d	18 ± 0.07c	23.67 ± 0.09d	24.67 ± 0.04d
*P*-value		< 0.01	< 0.01	< 0.01	< 0.01
*J. phoenicea*	80%	40 ± 0.11a	47 ± 0.07a	95.67 ± 0.04a	98.33 ± 0.03a
	60%	31 ± 0.08b	39 ± 0.19b	92.67 ± 0.02a	92.67 ± 0.06a
	30%	30 ± 0.29b	27 ± 0.01c	51.33 ± 0.13b	48.67 ± 0.16b
	20%	21 ± 0.32c	24 ± 0.05d	34.67 ± 0.16c	37.33 ± 0.11c
	10%	10 ± 0.01d	14 ± 0.05e	22 ± 0.01c	22.33 ± 0.27d
*P*-value^b^		< 0.01	< 0.01	< 0.01	< 0.01

Values are mean ± standard error of the mean for bioassay conducted in triplicates; ^a^Tukey’s Test, values followed by different superscripts are significantly different at *P* ≤ 0.05; ^b^Probabilities associated with individual F tests.

For *P. lentiscus* and *E. globulus*, statistical analysis showed a significant difference (*P* =  < 0.05) between the homogeneous group of the three concentrations C3, C4, and C5 and the group of C1 and C2. However, for *J. phoenicea* the only concentration that showed a significant effect on the inhibition of the growth of *C. gloeosporioides* was C5. When comparing all concentrations of the three extracts, we note that *E. globulus* provided the best decrease in fungal mycelial growth at all doses (Table 7).

Regarding *A. alternata*, the C5 concentration of *E. globulus* aqueous extract could inhibit the mycelia growth of the fungus with an inhibition percentage of 80%. However, the aqueous extract obtained from the species *P. lentiscus* didn’t show a promising result since it could only give an inhibition percentage of 50% with three different concentrations C3, C4, and C5. The statistical analysis clearly showed that in all studied species the C5 concentration significantly reduced the mycelial growth of A. alternate (*P* < 0.05). However, in the case of *P. lentiscus*, a significant difference was observed between C1 and C2. Likewise, we record a significant difference between C3, C4, and C5. When the fungistatic activity of the three extracts was compared, it was evident that the extract *J. phoenicea* was the least efficient in inhibiting the development of both fungi (*A. alternata* and *C. gloeosporioides*) (Table 7).

The fungus with an inhibition percentage of 80%. However, the aqueous extract obtained from the species *P. lentiscus* didn’t show a promising result since it could only give an inhibition percentage of 50% with three different concentrations C3, C4, and C5. The statistical analysis clearly showed that in all studied species the C5 concentration significantly reduced the mycelial growth of A. alternate (*P* < 0.05). However, in the case of *P. lentiscus*, a significant difference was observed between C1 and C2. Likewise, we record a significant difference between C3, C4, and C5. When the fungistatic activity of the three extracts was compared, it was evident that the extract *J. phoenicea* was the least efficient in inhibiting the development of both fungi (*A. alternata* and *C. gloeosporioides*) (Table 7).

### Minimum inhibitory concentration

*C. gloeosporioides* and *A. alternata* exhibited high MICs to *J. phoenicea* essential oil with MICs > 7.514 μg mL^−1^.*E. globulus* essential oil had the lowest activity against both phytopathogens (Table 8).

**Table 8:** Minimum inhibitory concentrations (MIC) of *E. globulus*, *P. lentiscus* and *J. phoenicea* essential oils against *C. gloeosporioides* and *A. alternata***.**

	MIC (µg mL^−1^)	
	*C. gloeosporioides*	*A. alternata*
*E. globulus*	5.103	5.489
*P. lentiscus*	5.615	6.427
*J. phoenicea*	7.658	7.514

MIC, Minimum inhibitory concentration.

### In vivo fungistatic activity assay

The evaluation of essential oils and aqueous extract’s effectiveness in controlling *A. alternata* and *C. gloeosporioides* on apple fruits showed that the lesion diameter (LD) and disease severity index (DSI) were significantly lower compared to positive controls (*P* < 0.01).Generally, a significant decrease in DSI and LD values was observed with augmentation in the concentration (Tables 9 and 10).

**Table 9:** Effect of preventive treatments of essential oils of *E. globulus, P. lentiscus* and *J. phoenicea* on the aggressiveness of *A. alternata* and *C. gloeosporioides* on apple fruits.

Treatments		Lesion diameter (mm)		Disease severity index (%)		Efficacy of treatment	
		*C. gloeosporioides*	*A. alternata*	*C. gloeosporioides*	*A. alternata*	*C. gloeosporioides*	*A. alternata*
Positive control		34.80 ± 0.47a^a^	34.20 ± 0.15a	96 ± 0.67a	96.83 ± 0.48a	HI	HI
Negative control		0 ± 0 h	0 ± 0 g	0 ± 0 g	0 ± 0 h	EE	EE
*E. globulus*	4 µl/ml	3.83 ± 0.57 g	3.18 ± 0.63f.	8 ± 0.99f.	4.83 ± 0.33 g	E	HE
	2 µl/ml	6 ± 0.5f.	5.9 ± 0.19f.	12 ± 0.89e	10.67 ± 0.18f.	E	E
	1 µl/ml	11.07 ± 0.45e	9.13 ± 0.21e	22.67 ± 0.57d	17.33 ± 0.15e	E	E
	0.5 µl/ml	13 ± 0.5d	13.08 ± 0.20d	26 ± 0.79d	25 ± 0.64d	I	E
	0.25 µl/ml	17.33 ± 0.28c	20 ± 0.45c	34.67 ± 0.57c	38.67 ± 0.15c	I	I
	0.125 µl/ml	24.17 ± 0.52b	29.42 ± 0.97b	52.5 ± 0.5b	61.67 ± 0.88b	HI	HI
*P*-value^*b*^		< 0.01	< 0.01	< 0.01	< 0.01	Nd	Nd
Positive control		37.03 ± 0.56a	39.73 ± 0.20a	99.5 ± 0.87a	92.5 ± 0.5a	HI	HI
Negative control		0 ± 0 g	0 ± 0 g	0 ± 0 g	0 ± 0f.	EE	EE
*P. lentiscus*	4 µl/ml	2.67 ± 0.29f.	1.43 ± 0.80 g	5.5 ± 0.5f.	2.5 ± 0.89ef	E	HE
	2 µl/ml	4.33 ± 0.28f.	3.4 ± 0.45f.	9 ± 0.79e	6.83 ± 0.78e	E	E
	1 µl/ml	6.67 ± 0.3e	6.73 ± 0.25e	15.5 ± 0.77d	13.17 ± 0.76d	E	E
	0.5 µl/ml	8.73 ± 0.32d	9.37 ± 0.75d	18.17 ± 0.06d	17.67 ± 0.58d	E	E
	0.25 µl/ml	14 ± 0.5c	11.5 ± 0.99c	28.5 ± 0.5c	23 ± 0.85c	I	E
	0.125 µl/ml	22.17 ± 0.52b	20.97 ± 0.50b	51.5 ± 0.60b	41 ± 0.61b	HI	I
*P*-value		< 0.01	< 0.01	< 0.01	< 0.01	Nd	Nd
Positive control		38.53 ± 0.95a	34.53 ± 0.64a	92.33 ± 0.63a	76.5 ± 0.60a	HI	HI
Negative control		0 ± 0f.	0 ± 0f.	0 ± 0f.	0 ± 0 g	EE	EE
*J. phoenicea*	4 µl/ml	0.83 ± 0.57f.	0.67 ± 0.28f.	1.83 ± 0.04ef	1.33 ± 0.57 g	HE	HE
	2 µl/ml	1.33 ± 0.04ef	6.87 ± 0.55e	3 ± 0.73ef	14 ± 0.55f.	HE	E
	1 µl/ml	3 ± 0.5e	13.5 ± 0.22d	6.33 ± 0.15e	27.5 ± 0.27e	E	I
	0.5 µl/ml	10.20 ± 0.53d	16.17 ± 0.76c	21.5 ± 0.73d	32.5 ± 0.32d	E	I
	0.25 µl/ml	14 ± 0.5c	18.17 ± 0.29bc	28 ± 0.96c	37.67 ± 0.52c	I	I
	0.125 µl/ml	19.5 ± 0.55b	20.33 ± 0.28b	41.83 ± 0.11b	44.67 ± 0.03b	I	I
*P*-value^b^		< 0.01	< 0.01	< 0.01	< 0.01	Nd	Nd

^a^Tukey’s Test, values followed by various superscripts differ significantly at *P* ≤ 0.05.^b^Probabilities associated with individual F tests. Data are the average of 27apple fruits per treatment and replicate (3 replicates). Means ± standard error.

**Table 10:** Effect of preventive treatments of aqueous extracts of *E. globulus, P. lentiscus*, and *J. phoenicea* on the aggressiveness of *A. alternata* and *C. gloeosporioides* on apple fruits.

Treatments		Lesion diameter (mm)		Disease severity index (%)		Efficacy of treatment	
		*C. gloeosporioides*	*A. alternate*	*C. gloeosporioides*	*A. alternate*	*C. gloeosporioides*	*A. alternata*
Positive control		37.80 ± 0.26a ^a^	39.23 ± 0.02a	99.50 ± 0.86a	98.50 ± 0.59a	HI	HI
Negative control		0 ± 0e	0 ± 0f.	0 ± 0f.	0 ± 0f.	EE	EE
*E. globulus*	80%	5.50 ± 0.86d	4.75 ± 0.17e	15 ± 0.66e	6.83 ± 0.75e	E	E
	60%	7.33 ± 0.60d	6.40 ± 0.43d	20.33 ± 0.88de	12 ± 0.73e	E	E
	30%	14.17 ± 0.30c	7.17 ± 0.29d	28.67 ± 0.13 cd	21 ± 0.64d	I	E
	20%	15.17 ± 0.44c	12.30 ± 0.34c	32.33 ± 0.62c	27.33 ± 1.12c	I	I
	10%	27.17 ± 1.43b	28.97 ± 0.53b	58.67 ± 1.08b	64.67 ± 0.16b	HI	HI
*P*-value^*b*^		< 0.01	< 0.01	< 0.01	< 0.01	Nd	Nd
Positive control		38.50 ± 0.59a	38.63 ± 1.13a	97 ± 0.96a	94.20 ± 0.19a	HI	HI
Negative control		0 ± 0e	0 ± 0f.	0 ± 0e	0 ± 0f.	EE	EE
*P. lentiscus*	80%	5 ± 0.18d	4.87 ± 0.51e	11.50 ± 0.76de	5 ± 0.22ef	E	HE
	60%	7.33 ± 0.57 cd	6 ± 0.66e	16.83 ± 0.76 cd	9.17 ± 0.28e	E	E
	30%	8.33 ± 0.60 cd	9.50 ± 0.50d	21.97 ± 0.36 cd	16.50 ± 0.77d	E	E
	20%	10.50 ± 0.32c	17.20 ± 0.54c	25.30 ± 0.80c	23.33 ± 0.62c	I	E
	10%	25.83 ± 1.03b	26.80 ± 0.52b	61.83 ± 0.82b	45 ± 1.09b	HI	HI
*P*-value		< 0.01	< 0.01	< 0.01	< 0.01	Nd	Nd
Positive control		39.87 ± 0.55a	37.80 ± 0.26a	96.17 ± 0.63a	92.83 ± 0.05a	HI	HI
Negative control		0 ± 0f.	0 ± 0 g	0 ± 0e	0 ± 0e	EE	EE
*J. phoenicea*	80%	3.83 ± 1.02ef	6.80 ± 0.34f.	7.50 ± 0.92de	6.33 ± 0.18e	E	E
	60%	5.50 ± 0.59d	11 ± 0.86e	11.33 ± 0.57de	17.67 ± 1.04d	E	E
	30%	7.17 ± 0.17d	19.80 ± 0.22d	16.50 ± 0.32 cd	29.33 ± 0.73c	E	I
	20%	12.87 ± 1.03c	22.63 ± 0.33c	26.33 ± 0.10c	38.50 ± 0.38c	I	I
	10%	24.83 ± 0.72b	29.37 ± 0.35b	51.67 ± 0.23b	51.67 ± 0.48b	HI	HI
*P*-value^b^		< 0.01	< 0.01	< 0.01	< 0.01	Nd	Nd

^a^Tukey’s Test, values followed by various superscripts differ significantly at *P* ≤ 0.05.^b^Probabilities associated with individual F tests. Data are the average of 27apple fruits per treatment and replicate (3 replicates).Means ± standard error.

Fruits treated separately with essential oils at a concentration of 4 µl/ml revealed a significant reduction in LD, as the values ranged from 0.83 mm (*J. phoenicea*) to 3.83 mm (*E. globulus* essential oil) for *C. gloeosporioides* (34.80 mm ≤ positive control ≤ 38.53 mm), and varied from to 0.67 mm (*J. phoenicea*) and 3.18 mm (*E. globulus*) for *A. alternata* (34.20 mm ≤ positive control ≤ 39.73 mm) (Table 9).The effect of essential oils on DSI was less observed at a concentration of 4 µl/ml, and the values ranged between1.83% (*J. phoenicea*) and 8% (*E. globulus*) for *C. gloeosporioides* (92.33% ≤ positive control ≤ 99.5%), and between 1.33% (*J. phoenicea*) and 4.83% (*E. globulus*) for *A. alternata* (76.5% ≤ positive control ≤ 96.83%) (Table 9). However, the apple fruits treated with essential oils at a concentration of 0.125 µl/mlshowed the highest LD and DSI (Table 9).

Aqueous extracts of *E. globulus, P. lentiscus,* and *J. phoenicea* at a concentration of 80% were significantly more effective in reducing the lesion diameter of *C. gloeosporioides* (3.83 and 5.50 mm for *J. phoenicea* and *E. globulus*, respectively) and *A. alternata* (4.75 and 6.80 mm for *E. globulus* and *J. phoenicea*, respectively) than other treatments (Table 10). In the same sense, the lowest DSI were obtained from the apple fruits treated with aqueous extracts at a concentration of 80% and the values ranged between 5% (*P. lentiscus*/*A. alternata*) and 15% (*E. globulus*/*C. gloeosporioides*) (Table 10).

The treatment effectiveness using essential oils and aqueous extracts of *E. globulus, P. lentiscus,* and *J. phoenicea* was variable (Tables 9 and 10). The treatment with essential oils at a concentration of 4 µl/ml showed its ability to protect the apple fruits against *A. alternata* and *C. gloeosporioides* (Table 9). The same results were obtained after treatments with aqueous extracts at a concentration of 80% (Table 10). Nevertheless, all treatments using essential oils at 0.125 µl/ml and aqueous extracts at 10% have shown their ineffectiveness in reducing the aggressively of *A. alternata* and *C. gloeosporioides*. Thus, the positive controls were highly sensitive to *A. alternata* and *C. gloeosporioides* attacks (Tables 9 and 10).

At high concentrations, essential oils have a strong inhibiting effect on the growth potential of *A. alternata* and *C. gloeosporioides* reaching up to 88.98%. Similarly, the protective potential was the highest in apple fruits treated separately with essential oils at 4 µl/ml (> 91.67%) (Table 11).

**Table 11:** Effect of preventive treatments of essential oils of *E. globulus*, *P. lentiscus*, and *J. phoenicea* on protective and inhibitory growth potential on apple fruits.

Treatments		Inhibitory growth potential (%)		Protective potential (%)	
		*C. gloeosporioides*	*A. alternata*	*C. gloeosporioides*	*A. alternata*
*E. globulus*	4 µl/ml	88.98 ± 0.65a ^a^	90.71 ± 0.84a	91.67 ± 0.04a	95.01 ± 0.32a
	2 µl/ml	82.76 ± 0.43b	82.75 ± 0.58b	87.5 ± 0.44b	88.98 ± 0.19b
	1 µl/ml	68.20 ± 0.29c	73.30 ± 0.60c	76.39 ± 0.60c	82.1 ± 0.33c
	0.5 µl/ml	62.64 ± 0.43d	61.76 ± 0.55d	72.92 ± 0.41d	74.18 ± 0.73d
	0.25 µl/ml	50.19 ± 0.82e	41.53 ± 0.33e	63.89 ± 0.66e	60.07 ± 0.23e
	0.125 µl/ml	30.56 ± 0.38f.	13.98 ± 0.85f.	45.31 ± 0.62f.	36.32 ± 0.98f.
*P*-value^*b*^		< 0.01	< 0.01	< 0.01	< 0.01
*P. lentiscus*	4 µl/ml	92.80 ± 0.77a	96.39 ± 0.03a	94.47 ± 0.55a	97.3 ± 0.93a
	2 µl/ml	88.30 ± 0.80b	91.44 ± 0.15b	90.95 ± 0.52a	92.61 ± 0.82b
	1 µl/ml	82 ± 0.70c	83.05 ± 0.63c	84.42 ± 0.11b	85.77 ± 0.88c
	0.5 µl/ml	76.42 ± 0.68d	76.43 ± 0.18d	81.74 ± 0.70b	80.90 ± 0.62d
	0.25 µl/ml	62.20 ± 0.35e	71.06 ± 0.51e	71.36 ± 0.50c	75.14 ± 0.16e
	0.125 µl/ml	40.14 ± 0.12f.	47.23 ± 0.88f.	48.24 ± 0.62d	55.68 ± 0.89f.
*P*-value		< 0.01	< 0.01	< 0.01	< 0.01
*J. phoenicea*	4 µl/ml	97.84 ± 0.49a	98.07 ± 0.84a	98.01 ± 0.12a	98.26 ± 0.75a
	2 µl/ml	96.54 ± 0.70ab	80.12 ± 0.60b	96.75 ± 0.87a	81.7 ± 0.37b
	1 µl/ml	92.21 ± 0.29b	60.91 ± 0.79c	93.14 ± 0.25a	64.05 ± 0.58c
	0.5 µl/ml	73.53 ± 0.99c	53.18 ± 0.21d	76.71 ± 0.87b	57.52 ± 0.72c
	0.25 µl/ml	63.67 ± 0.30d	47.39 ± 0.83e	69.68 ± 0.08c	50.76 ± 0.99d
	0.125 µl/ml	49.39 ± 0.59e	41.12 ± 0.88f.	54.69 ± 0.61d	41.61 ± 0.57e
*P*-value^b^		< 0.01	< 0.01	< 0.01	< 0.01

^a^Tukey’s Test, values followed by various superscripts differ significantly at *P* ≤ 0.05.^b^Probabilities associated with individual F tests. Data are the average of 27apple fruits per treatment and replicate (3 replicates). Means ± standard error.

Aqueous extracts exert potent and long-lasting effects on *A. alternata* and *C. gloeosporioides* growth with inhibition rates above85.45% (for inhibitory growth potential) and 84.92% (for protective potential) (Table 12).

**Table 12:** Effect of preventive treatments of aqueous extracts of *E. globulus, P. lentiscus*, and *J. phoenicea* on protective and inhibitory growth potential on apple fruits.

Treatments		Inhibitory growth potential (%)		Protective potential (%)	
		*C. gloeosporioides*	*A. alternate*	*C. gloeosporioides*	*A. alternata*
*E. globulus*	80%	85.45 ± 0.29a^a^	87.88 ± 0.44a	84.92 ± 0.03a	93.06 ± 0.80a
	60%	80.60 ± 0.25a	83.69 ± 0.11b	79.56 ± 0.12ab	87.82 ± 0.76a
	30%	62.52 ± 1.08b	81.73 ± 0.73c	71.19 ± 0.15b	78.68 ± 0.68b
	20%	59.88 ± 0.81b	68.65 ± 0.88d	67.50 ± 0.64b	72.25 ± 0.70b
	10%	28.13 ± 0.78c	26.17 ± 0.28e	41.04 ± 0.13c	34.35 ± 0.23c
*P*-value^*b*^		< 0.01	< 0.01	< 0.01	< 0.01
*P. lentiscus*	80%	87.01 ± 0.66a	87.40 ± 0.42a	88.14 ± 0.97a	94.69 ± 0.15a
	60%	80.95 ± 0.49ab	84.47 ± 0.29a	82.65 ± 0.99a	90.27 ± 0.19a
	30%	78.35 ± 0.17ab	75.41 ± 0.29b	77.35 ± 0.59a	82.48 ± 0.07b
	20%	72.73 ± 0.19b	55.48 ± 0.98c	73.92 ± 1.01a	75.23 ± 0.90b
	10%	32.90 ± 1.07c	30.63 ± 0.34d	36.25 ± 0.13b	52.23 ± 0.31c
*P*-value		< 0.01	< 0.01	< 0.01	< 0.01
*J. phoenicea*	80%	90.38 ± 0.68a	82.01 ± 0.91a	92.20 ± 0.12a	93.18 ± 0.24a
	60%	86.20 ± 0.51a	70.90 ± 0.29b	88.21 ± 0.79ab	80.97 ± 0.35a
	30%	82.02 ± 0.96a	47.62 ± 0.53c	82.84 ± 0.65ab	68.40 ± 0.49b
	20%	67.73 ± 1.08b	40.12 ± 0.85d	72.62 ± 0.31b	58.53 ± 0.87b
	10%	37.71 ± 0.85c	22.31 ± 0.92e	46.27 ± 0.64c	44.34 ± 0.21c
*P*-value^b^		< 0.01	< 0.01	< 0.01	< 0.01

^a^Tukey’s Test, values followed by various superscripts differ significantly at *P* ≤ 0.05.^b^Probabilities associated with individual F tests. Data are the average of 27 apple fruits per treatment and replicate (3 replicates). Means ± standard error.

## Discussion

Investigating numerous organoleptic and physicochemical properties enables us to better understand the usefulness of plant oils in daily life^40^. These characteristics, which offer a foundational assessment of an oil's fitness for use, are largely responsible for its commercial significance^41,42^.

The acid index of oil is a crucial physicochemical characteristic index that is used to assess its quality, age, digestibility, and appropriateness for industrial applications like paints^34^. This index is used to determine how much of the oil's glycerides have been broken down by lipase and other physical elements like light and heat^43^.

The refractive index represents the degree to which a light beam is deflected when it moves from one transparent medium to another. It increases, as there are more carbon atoms and as chains get longer. The refractive index indicates that the sample may contain an unsaturated long carbon chain^44^. Measurements of the refractive index are particularly useful for determining the purity of volatile and fixed oils^45^.

The obtained average extraction yield, in this work of *E. globulus*, is higher than that achieved by some researchers like Kumar et al.^46^ and Sameza et al.^47^, in which they found yields of 0.72 and 0.82%, respectively. Recent studies show that the yields of essential oils obtained from several species of Eucalyptus vary between 1.2 and 3%^48^. This variation between essential oil yields is probably attributed to the difference in the age of the leaves^49^ or to the time extraction of essential oils^46^.

The comparison of the essential oil yield of *J. phoenicea* revealed that it is equivalent to those produced by previous works in Tunisia, which range from 0.5 to 0.9%^50,51^. Lower yields of (0.39; 0.21; and 0.30) have been recorded in studies conducted in other Mediterranean nations such as Algeria, Greece, and Spain^52^. The yield of essential oil extracted from *P. lentiscus* was lower than that obtained by Zrira^53^ and Trabelsi et al.^54^ which is between 0.2 and 0.4% and higher than that obtained by Luigia et al.^55^ estimated at 0.07%. These differences in results can be explained by the fact that the yields of essential oils are influenced by several factors during their extraction: either factors related to the plant (species, variety, chemical race, geographical origin, etc.) or factors related to the experimental conditions (extraction process, extraction time, the part of the plant used, etc.)^56,57^. Moreover, these authors have shown that the location and duration used for drying leaf samples highly influence the yield of essential oil.

When comparing the organoleptic features of *J. phoenicea* to those found by Bouchenak et al.^58^, results revealed some conformity with a slight difference in density (ranging from 0.850 to 0.872).

Despite the high TPC on *E. globulus* obtained in this study, Zin et al.^59^ reported higher contents by the order of 432.63 μg EAG/mg DW).

Results obtained with *P. lentiscus* are close to those of Ebrahimzadeh et al.^60^ who obtained (289.5 mg EAG/g DW) of TPC. However, our result is higher than those reported by Atmani et al.^61^ (136.25 ± 18.9 mg EAG/g DW). TPCs on *J. phoenicea* are found superior to those determined in the studies of Soltani et al.^62^ with a content of 114.00 mg EAG/g DW and Hayouni et al.^63^ with a content of 167 mg EAG/g DW, and Keskes et al.^64^ with a content of 168 mg EAG/g DW.

Our results showed several phytochemicals including phenolic diterpenes, flavonoids, organic compounds, and phenolic acids that are known to be beneficial sources of natural antioxidants and could be isolated for a variety of medicinal, cosmetic, or agro-industrial applications^65^.

When comparing our *J. phoenicea* extract results with those cited in the literature, we note that according to Soltani et al.^62^, the flavonoid concentration of leaf extracts was quite high, with a value of 140.10 mg EC/g DW and Laouar et al.^66^ have been recorded extremely low values (l2.09 mg EQ/g DW). Studies by Zaouali et al.^67^ found a higher flavonoid concentration in the extract of the dry leaves of *Lentiscus* species (47.5 mg EQ/g DW). On the other hand, the flavonoid concentrations measured in *E. globulus* extracts are significantly higher than those reported in previous studies by Nicoláset al.^68^.

According to Atmani et al.^61^, the leaves of *P. lentiscus* have an anti-free radical activity (DPPH) of 93%, which is significantly higher than that of the current study. Similarly, extracts of *E. globulus* had significant percentages of free radical catching DPPH (97.63 percent and 76.2 percent, respectively)^69^. However, the obtained results by Medini et al.^70^ on *J. phoenicea* harvested in Tunisia, have an antioxidant activity of the methanolic extracts, which is more important, compared to this work, their activity varies from 72.15 to 95.89%.

The HPLC phytochemical analysis revealed that the extract of *E. globulus* has significant antioxidant activity and a high concentration of phenols and flavonoids. These results are confirmed by those of Boulekbache-Makhlouf et al.^71^. However, catechins are the main flavonoid found in high amounts in *J. phoenicea* extract.

Leaves of several *Eucalyptus* species have shown significant biological activities such as antimicrobial, fungistatic, insecticidal, herbicide, acaricidal, and nematicidal^72^.

Moreover, crude extracts of *Pistacia vera*, *P. terebinthus*, and *P. lentiscus* leaves prevented the development of *Pythiumultimum* and *Rhizoctoniasolani*^73^.

Several studies have also reported that the essential oil from the aerial parts of *P. lentiscus* has significant antifungaland antibacterial properties^73,74^. Indeed, El Idrissi et al. 2016^75^ reported that the essential oil of *P. lentiscus* inhibited the development of *R. solani*, *F. sambucinum*, and *Candida albicans* more than that of *Penicillium*.

Treatment with aqueous extracts and essential oils showed a valuable positive effect in laboratory conditions, confirming the findings of previous researchers. Indeed, Zhou et al.^76^ documented that the essential oils of *Eucalyptus* spp. effectively inhibited the growth of *A. alternata* and *C. gloeosporioides* in vitro. Ghaffar et al.^77^ and Salem et al.^78^ revealed the effectiveness of *Eucalyptus* spp. oils in controlling *Alternaria* spp. and *Colletotrichum* spp. under in vitro conditions. More so, Pedrotti et al.^79^ reported the ability of *Eucalyptus* sp. to reduce the mycelial growth and colonization of *A. alternaria*, and to inhibit the sporulation and spore germination. El Idrissi et al.^75^ documented that *P. lentiscus* oils showed the strongest antibacterial and antibacterial activities under laboratory conditions. Kordali et al.^73^ depicted that *Pistacia* spp. oils greatly lowered mycelial growth and spore germination. As stated by Pepeljnjak et al.^80^; Sati and Joshi 2010^81^ and Bais et al.^82^, treatment with aqueous extracts and essential oils of *Juniperus* spp. significantly decreased the fungi mycelial growth.

This study proved that essential oils (at 4 µl/ml) and aqueous extracts (at 80%) of *E. globulus, P. lentiscus,*and*J. phoenicea* have an effective bio-fungicides potential against *A. alternata* and *C. gloeosporioides* on apple fruits. Similar to our results, previous reports confirmed the efficacy of aqueous extracts and essential oils against the pathogenicity of *A. alternata* and *C. gloeosporioides*^37,83^. Ikeura et al.^84^ illustrated the effectiveness of aqueous extracts and essential oils in controlling *Penicillium expansum* on apple fruits. Steglińska et al.^85^ documented that plant extracts of *Eucalyptus* sp. effectively inhibited the growth of *Colletotrichum* spp. and *Alternaria* spp. under in vivo conditions.

The antifungal potential of several plant extracts was linked to their phytochemical composition and bioactive compounds like phenolic acids. Phenolic can trigger the plant's natural defense responses, leading to increased production of antifungal compounds and strengthening the plant's cell walls^2^. The hydroxyl groups of phenolics disrupt the cell membranes of *Colletotrichum* spp. and *Alternaria* spp., causing leakage of vital cellular components and ultimately leading to cell death^7^. Moreover, some phenolics can hinder the function of key enzymes involved in the metabolism of *Colletotrichum* spp. and *Alternaria* spp., disrupting their growth and reproduction^3,46^.

The phytochemical composition of essential oil of *Eucalyptus globulus* exhibits a remarkable multi-layered defense against fungal pathogens. Monoterpenes (1,8-cineole, α-pinene, and limonene) dominate the composition^86,87^. These compounds display potent radical scavenging activity, effectively neutralizing reactive oxygen species (ROS) that can damage fungal cell membranes and disrupt vital cellular processes^88^. This antioxidant shield directly protects the fungal cells from oxidative stress, potentially weakening their virulence^87,89^. In addition to antioxidant defenses, the essential oil has powerful fungicidal activity^90^. Limonene and α-pinene cause disruption of fungal cell membranes through their lipophilic properties and lead to cell leakage and collapse^91^. 1,8-cineole inhibits fungal growth and spore germination, effectively cutting off the reproductive pathways and preventing further colonization^87,92^. Phenolic compounds exemplified by α-terpineol, add another layer of defense with their inherent antifungal properties, further bolstering the overall efficacy against fungal pathogens^93^.

The essential oil extracted from *Pistacia lentiscus* exhibits a fascinating interplay between its phytochemical composition, antioxidant activity, and potent fungicidal effects, ultimately creating a multi-pronged defense against phytopathogens^67^. Monoterpenes possess antioxidant properties, particularly myrcene, and α-terpineol, which scavenge harmful ROS that can damage fungal cell membranes and disrupt vital processes^94^. This protective shield indirectly weakens the phytopathogens, making them more susceptible to other essential oils^56,57^. The lipophilic nature of α-pinene and limonene allows them to infiltrate and disrupt fungal cell membranes, causing leakage and ultimately leading to cell collapse^95^. Furthermore, sesquiterpenes such as α-caryophyllene inhibit fungal enzymes and metabolic pathways^56,57,96^. The antioxidant action of myrcene and α-terpineol weakens the phytopathogen's defenses, making them more susceptible to membranous assaults^97^. This interplay amplifies the individual potencies of each compound, creating a robust and multifaceted defense system. Additionally, the presence of phenolic compounds like gallic acid and its derivatives can contribute to antifungal activity through their direct interaction with fungal membranes and proteins^56,57,98,99^.

*Juniperus phoenicea* essential oil boasts a rich of volatile compounds, each playing a crucial role in its antifungal arsenal. *Juniperus phoenicea* essential oil boasts a diverse cast of monoterpenes like α-pinene, sabinene, and β-pinene. Their lipophilic nature allows infiltrating and disrupting fungal cell membranes, causing leakage and ultimately celling death^100,101^. Sesquiterpenes (α-cedrene and β-caryophyllene) inhibit crucial fungal enzymes and metabolic pathways^102^. Phenolic compounds (thymol and carvacrol) are wielding their potent antioxidant properties to neutralize ROS generated by phytopathogens. These ROS can damage fungal cells and weaken their defenses, making them more susceptible to the membranous assaults of the monoterpenes and sesquiterpenes^100^. In an accordant study, the potential of thymol and carvacrol enhanced the disruption of fungal membranes by monoterpenes, acting as synergistic co-solvents^103^.

The true masterpiece lies in the intricate synergy between these components. The antioxidant shield of these essential oils provided by phenolic compounds weakens fungal defenses, allowing monoterpenes and sesquiterpenes to suppress pathogens. This combined assault amplifies the individual powers of each compound, creating a robust and multifaceted defense system^56,57,103^.

## Conclusion

From the aforementioned results, the present investigation shows that the aqueous and EO extract of *E. globulus* leaves exhibit good efficiency in inhibiting the growth and spore germination of tested pathogenic fungi. These extracts have a brilliant future in *C. gloeosporioides* and *A. alternata* management to substitute synthetic fungicides. Due to the limited number of commercially natural compounds, it is of great interest to deepen our knowledge about the molecular mechanisms and discover new bio-pesticide and bio-stimulant agents. In addition, the impact of plant extracts application on the consumer’s acceptability and fruit sensory characteristics need to be carefully considered. These obtained results from laboratory experiments, could be supplemented also by other in vivo studies, both in controlled greenhouse conditions and in open fields to practically evaluate the use of these extracts in the frame of an Integrated Crop Management System and introduce them into practical use in preventive conservation.

## Data Availability

The datasets used and/or analyzed during the current study available from the corresponding author on reasonable request.
